# The days we never forget: Flashbulb memories across the life span in Alzheimer’s disease

**DOI:** 10.3758/s13421-024-01558-z

**Published:** 2024-04-16

**Authors:** Katrine W. Rasmussen, Marie Kirk, Susanne B. Overgaard, Dorthe Berntsen

**Affiliations:** Center on Autobiographical Memory Research, Department of Psychology and Behavioral Sciences, Aarhus University, Bartholins Allé 9, 8000 Aarhus C, Denmark

**Keywords:** Alzheimer’s disease, Autobiographical memory, Flashbulb memory, Temporal distribution, Reminiscence bump

## Abstract

Alzheimer’s disease (AD) is characterized by autobiographical memory deficits, with the ability to retrieve episodic-rich memories being particularly affected. Here, we investigated the influence of AD on a specific subtype of episodic memories known as flashbulb memories (i.e., the ability to remember the personal circumstances for the reception of important news events). We examined the frequency, characteristics, and the temporal distribution of flashbulb memories across the life span. To this aim, 28 older adults diagnosed with AD and a matched sample of 29 healthy older controls were probed for flashbulb memories for two historical events from each decade of their lives. They also estimated the subjective degree of reexperiencing for the memories reported. AD participants showed impaired access to flashbulb memories, the frequency of reported memories being lower than for healthy older adults. However, qualitative aspects of AD participants’ flashbulb memories were quite similar to those of the controls, as no group differences were obtained with respect to the canonical categories or degree of reexperience. AD participants’ flashbulb memories clustered during the early years of their life, consistent with a reminiscence bump, whereas healthy controls also reported memories dated to later lifetime periods. Our results suggest that probing for personal memories of important public events may serve as a powerful cue for detailed episodic memories in AD.

One of the earliest manifestations of Alzheimer’s disease (AD) is episodic memory loss, which has largely been attributed to medial temporal lobe (MTL) atrophy (Tromp et al., 2015). Episodic memory refers to memory for personal experienced events and their spatiotemporal context and is accompanied by a subjective sense of reexperiencing the event, known as autonoetic consciousness (Tulving, 1985). Although, episodic memory deficits have been consistently documented, with individuals with AD experiencing difficulties recalling contextual details (e.g., Greene et al., 1995; Hou et al., 2005; Ivanoiu et al., 2006; Leyhe et al., 2009) and an impaired capacity to mentally reexperience past events (e.g., Irish et al., 2011; Piolino et al., 2003), less is known about a particular subtype of episodic memories, known as *flashbulb memories* (FBMs), and the degree to which they are disrupted by the disease.

The term FBM was introduced by Brown and Kulik (1977) to account for vivid, detailed, and long-lasting memories for the circumstances in which one first learn about surprising and highly emotional public or personal events. Prominent examples include the assassination of President Kennedy, the Space Shuttle Challenger explosion, and the September 11 terrorist attacks (Luminet & Curci, 2009). A core feature of FBMs is the recall of contextual and often irrelevant information, including where one was located and what one was doing, when receiving the news, or perceptual details about the weather or clothes worn at the time. Although FBMs are prone to forgetting and distortions, people often retain these memories over long retention intervals, with a strong sense of recollection, that is, with a high degree of vividness, sense of reliving and confidence (e.g., Berntsen & Thomsen, 2005; Conway et al., 2009; Talarico & Rubin, 2003). Surprisingly, relatively little is known as to how AD affects the ability to retain FBMs, despite some of their defining characteristics (e.g., episodic details) being especially affected by the disease.

So far, studies examining FBM in AD have focused on anterograde memory for emotional public events (Budson et al., 2004, 2007; Çebi et al., 2020; El Haj et al., 2016; Ikeda et al., 1998; Mori et al., 1999; Thompson et al., 2004; for reviews, see Broster et al., 2012; Tat et al., 2018), and not examined retrograde amnesia for FBM events. The research on anterograde amnesia provides evidence that individuals with AD retain some ability to encode and consolidate personal memories of public events. For instance, Ikeda et al. (1998) showed that 86.3% of AD participants remembered the 1995 Kobe earthquake after 2 months, compared with only 31.4% remembering a distinctive, but less emotional event (an fMRI examination) that occurred during the same period. However, whether these memories can be conceptualized as FBMs remains debated (for reviews, see Broster et al., 2012; Tat et al., 2018). Budson et al. (2007) examined FBM consistency of the September 11 attacks across time in AD and healthy aging. Compared with controls, AD participants showed impaired recall in the weeks following the attack and more rapid forgetting between the initial assessment and a 3-month follow-up. However, like controls, AD participants’ personal recall remained relatively stable from 3 months to 1 year after the event. The authors speculated that the primary memory deficit in AD was attributable to impaired encoding and more rapid forgetting, but that once memories were consolidated, forgetting occurred at similar rates as in healthy aging (Budson et al., 2007). This suggests that people with AD can retain personal memories of important public events over long intervals (see Thompson et al., 2004, for a similar conclusion).

Surprisingly, FBMs for events that have occurred prior to the onset of disease have yet to be examined in AD. In other words, no studies have examined retrograde amnesia for FBM events in dementia. This is surprising, since studying FBMs encoded before the onset of brain pathology may provide important insights into how AD impacts the retrieval of highly emotional events by excluding possible contamination from deficient encoding. The present study aimed to address this gap in the literature by investigating how AD affects retrograde memory for the reception context of important public events across the life span. Moreover, by examining FBMs for public events that occurred across the participants’ life span, the present study can help to clarify whether memories for remote events may show a relative sparing in AD.

In healthy older individuals, emotional events are typically remembered better and are less likely to be forgotten than more mundane events (e.g., Comblain et al., 2005; St. Jacques & Levine, 2007), and some studies suggest that FBMs may be unaffected by the age-related decline usually reported in episodic memory (e.g., Davidson et al., 2006; Davidson & Glisky, 2002; however, see Tekcan & Peynircioglu, 2002, for a different results). Research shows that emotional enhancement of memory is modulated by amygdala recruitment both at encoding (e.g., Adolphs et al., 1997; Cahill et al., 1996; Canli et al., 2000) and retrieval (Dolcos et al., 2017). When it comes to retrieval, neuroimaging studies have linked amygdala activation to increased emotional intensity, recall of contextual details, and sense of reexperiencing the event (Botzung et al., 2010; Sharot et al., 2004; Smith et al., 2005; Buchanan, 2007), and individuals with amygdala damage show impaired recall for emotional events including FBMs (e.g., Buchanan et al., 2006; Spanhel et al., 2018).

In contrast, little is known about retrograde memory for emotional autobiographical events in AD, despite amygdala atrophy being pronounced from the early stages of the disease (e.g., Basso et al., 2006; Horinek et al., 2007; Poulin et al., 2011). To our knowledge, only one study has examined autobiographical memories of emotional events in AD. Philippi et al. (2015) found that while AD participants recalled fewer emotional memories than controls, memory specificity did not differ between groups, indicating that emotions at the time of the event may increase subsequent recall of details in AD. However, the distinction between neutral and emotional memories was based on participants’ retrospective evaluation of their reaction at the time of the event and thus may have been affected by deficient recall.

Moreover, these findings may not generalize to FBM, as FBMs differ from other emotional memories in important ways. In contrast to emotional memories of personal events, the prototypical FBM involves public events, which means that social and cultural factors, including rehearsal and commemoration processes, may influence the formation and maintenance of FBMs differently than private memories (e.g., Berntsen, 2009; Hirst & Meksin, 2018; Wang & Aydin, 2018). For instance, Rasmussen and Berntsen (2009) conducted a comparison of five types of autobiographical memories (i.e., most positive memory, most negative memory, most frequent involuntary memory, most vivid FBM, and a control memory from the previous week). FBMs were more often shared with other people compared with other types of autobiographical memories, and they were rated significantly higher on social function. This finding aligns with arguments about the collective nature of FBMs and their importance to social identity (e.g., Neisser, 1982; Berntsen, 2009; Hirst & Phelps, 2016; Luminet & Curci, 2009). These claims are further supported by evidence that the likelihood for developing FBMs for public events are influenced by social group membership (e.g., gender, religion, nationality) (Talarico et al., 2019; see Berntsen, 2018 for a review). For example, Berntsen and Thomsen (2005) examined FBMs in relation to the occupation and liberation of Denmark during World War II and found that ties to the Danish resistance movement affected both the accuracy and clarity of memories, and Curci et al. (2001) observed more FBMs related to the death of President Mitterrand among French compared with Belgian citizens. Importantly, several studies have highlighted the importance of rehearsal for the long-term maintenance of FBMs (e.g., Berntsen & Thomsen, 2005; Bohn & Berntsen, 2007; Talarico & Rubin, 2003), with rehearsal also being linked to better memory preservation in AD (Müller et al., 2016). While most work has focused on highly surprising negative events, FBMs can also be formed for positive and/or expected events (e.g., Berntsen & Thomsen, 2005; Bohn & Berntsen, 2007; Curci & Luminet, 2009; Kraha & Boals, 2014; Tekcan, 2001). One goal of the present study was to examine both the frequency with which people with AD retain FBMs of public events and the qualitative aspects of FBMs including the level of detail and the phenomenological experience.

Studying FBMs in AD can also help to clarify the temporal extension of retrograde memory deficits in this population. Some studies have found that impairments follow a temporal gradient (Ribot, 1881), with memories for more remote events being better preserved than recent once, while others have reported flat ungraded impairments, or only a gradient for episodic memory or personal semantics (see Kirk & Berntsen, 2018; Kopelman & Bright, 2012, for reviews). A similar disagreement exists for public event knowledge (Kopelman, 1989; Leplow et al., 1997; Wilson et al., 1981; see Meeter et al., 2007, for a review). The presence of a temporal gradient seems to support the standard consolidation theory (Alvarez & Squire, 1994; Squire & Alvarez, 1995), which stipulates that the hippocampus plays a time-limited role in the storage of memories, where upon their retrieval become independent of the MTL. In contrast, ungraded episodic memory impairments across the life span are consistent with multiple trace theory (Gilboa & Moscovitch, 2021; Moscovitch & Gilboa, 2021; Nadel & Moscovitch, 1997), which argues that perceptually rich specific memories continue to depend on MTL structures. Here, the better preservation of remote memories sometimes reported have been explained by a process of semantization.

More recently, a different explanation has been offered to account for the finding that people with AD seem to show a relative preservation of memories from young adulthood, proposing that these findings may reflect the presences of a reminiscence bump (e.g., Berntsen et al., 2022; Kirk & Berntsen, 2018; Rasmussen & Berntsen, 2023; see Kopelman, 2019, for a review). Together with childhood amnesia (a scarcity of memories from the earliest years of life), the reminiscence bump represents one of the most robust findings in autobiographical memory and refers to the phenomenon that people recall a disproportionately high number of memories that occurred between the ages of 10 and 30 (Fitzgerald, 1988; Rubin & Schulkind, 1997; for a review, see Koppel & Berntsen, 2015; Munawar et al., 2018). The bump has also been demonstrated for important public events (e.g., Janssen et al., 2008; Rubin et al., 1998). In a now classic study, Schuman and Scott (1989) found generational effects in collective memories of important public events, showing that people were more likely to report events that took place during their teens or early 20s. However, findings are mixed with some public events demonstrating a bump, while others deviate from this temporal pattern (for reviews, see Koppel, 2013; Tekcan et al., 2017), likely reflecting an interaction with the historical significance of the event.

There is also some evidence to suggest that FBMs show a reminiscence bump. For example, Tekcan and Peynircioglu (2002) examined FBMs for two remote historical events in a group of elderly Turks and found that the formation of FBMs was associated with age at the time of the event. People who were in the bump period at the time of the events were more likely to form FBMs than people who were younger at the time (6 to 10 years), likely due to the importance of the event not being fully appreciated by the younger age group. Denver et al. (2010) reported that when older adults were free to recall FBMs from their lives, they produced a clear reminiscence bump, and that FBMs from the bump period were more detailed than FBMs of a more recent event (September 11). Accordingly, a second goal of this study was to explore the temporal distribution of FBMs in AD.

## The present study

The present study aimed to explore retrograde memory for the reception context of important public events across the life span in a sample of individuals diagnosed with AD and matched healthy controls (HCs). More specifically, our investigation centered on three aspects that will be outlined below: (1) the frequency with which individuals with AD retain FBMs of public events; (2) the quality of FBMs including their level of detail and degree of reliving; and (3) the temporal distribution, including whether a temporal gradient and/or a reminiscence bump can be observed for FBMs in AD. To address these questions, we examined narratives of how participants first learned about a series of public events spanning from the 1930s to the 2010s. We were not interested in accessing memory accuracy, but rather the long-term retention and characteristics of FBMs.

The first question concerned the frequency of FBMs in people with AD and HCs. Here, we aimed to examine the overall frequency of FBMs based on the probed events, as well as the frequency of FBMs for each individual event. To date, studies examining FBMs in AD have focused on memory for a single event (e.g., September 11 attacks). This strategy is complicated by the fact that some public events for various reasons are more likely to lead to the formation and maintenance of FBMs (Luminet & Curci, 2009; Rice et al., 2018). To overcome this problem in the present study, we probed for FBMs for two historical events from each decade of participants’ lives, including both positive and negative events. We hypothesized that AD participants would report fewer FBMs compared with HCs. However, we also expected some events to be more likely to result in the formation of FBMs, with the two groups showing a similar pattern across events.

The second question involved the effect of AD on the phenomenological qualities of FBMs. Here, we focused on two defining aspects of FBM—namely, contextual details and the subjective sense of reliving. Different conceptualizations of FBMs have been used to examine anterograde memory performance for the reception context in AD, mirroring the general FBM literature (Kizilöz & Tekcan, 2013). In the present study, we examined FBMs based on Brown and Kulik’s (1977) six canonical categories (place, ongoing activity, source, own affect, affect in others, and aftermath), as well as four additional categories (time, others present, activity before, other details) identified in the FBM literature (e.g., Kizilöz & Tekcan, 2013; Thomsen & Berntsen, 2003), and we assessed the subjective sense of reexperiencing the event. Two predictions could be made based on the literature. On the one hand, we expected that AD participants would exhibit compromised retrieval of contextual details and reduced sense of reliving, in line with observations from studies using standard episodic memory measures in this population (e.g., Irish et al., 2011; Piolino et al., 2003). Alternatively, we expected the AD group to demonstrate preserved recall of contextual detail, due to the residual enhancement effect of emotion on the phenomenological aspects of memory (Philippi et al., 2015), as well as potential effects of private and collective rehearsal. This might also encompass enhanced sense of reexperience.

The third question concerned the temporal distribution of FBMs in AD. Because we systematically probed for two FBMs from each decade participants had lived through, we were able to provide unique data regarding the pattern of retrievals across the life span and observe a reminiscence bump, as well as a period of childhood amnesia, if such were present in the data. Studies on the reminiscence bump in AD for autobiographical memories in general (e.g., Berntsen et al., 2022; Rasmussen & Berntsen, 2023) would suggest a predominance of memories from childhood and young adulthood, followed by a steep decline of memories after the age of 30. Here, we sought to investigate if this also applied to the retrieval of FBMs.

## Method

### Participants

**Fig. 1:**
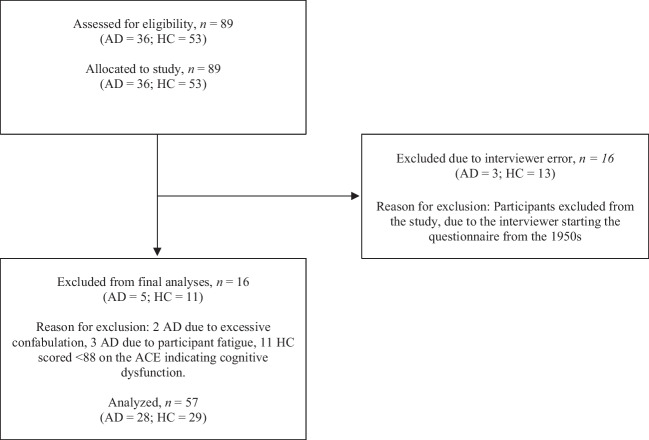
Flowchart showing attrition of participants

Of the 89 AD and HC participants who completed the flashbulb questionnaire, the final sample included 57 participants: 28 AD participants (14 female, 14 male) and 29 HC participants (14 female, 15 male); see Fig. 1 for an overview. Our sample size was determined based on previous research that compared autobiographical recall across the life span in AD (e.g., Berntsen et al., 2022; Philippi et al., 2015; Rasmussen & Berntsen, 2023) and a power analysis using G*Power (Version 3.1.9.2; Faul et al., 2007), with the following parameters: alpha = 0.05, power = 0.80, and effect size *d* = 0.80. All AD participants had received a diagnosis of “probably AD” at a hospital-based memory clinic in Denmark, based on general medical, neurological, and neuropsychological examination and according to the international guidelines by the National Institute on Aging and Alzheimer’s Association clinical criteria (McKhann et al., 2011). The HC participants were matched for age, gender, and education (see Table 1). Participants had no history of prior neurological problems, psychiatric disorders, or alcohol abuse. HC participants were also excluded from the final analyzes if they obtained a score <88 on the Addenbrooke’s Cognitive Examination (ACE; Mathuranath et al., 2000), as this indicates cognitive impairment. Both groups of participants demonstrated normal or corrected vision and hearing and were all Danish nationals. AD participants were recruited from municipality activity centers and local residential homes in Aarhus and Horsens, Denmark. HC participants were recruited from local senior’s organizations, and from the research center’s participant database. The study was approved by the Central Denmark Region Committees on Health Research Ethics, and all participants provided informed consent prior to participating in the study.

**Table 1:** Descriptive statistics and Welch *t*-test results for age, education, cognitive ability, and executive functioning

	Alzheimer (*n* = 28)		Control (*n* = 29)			
	*M*	*SD*	*M*	*SD*	*t*(*df*)	CI
Age (years)	80.82	6.18	79.72	6.23	*t*(55) = .647, *p* = .521	[−2.30, 4.50]
Education (years)	11.54	3.76	11.84	3.05	*t*(52) = −.340, *p* = .735	[−2.13, 1.51]
GDS/15	2.42	2.34	1.38	1.40	*t*(40) = 1.982, *p* = .054	[−0.02, 2.11]
MMSE/30	21.21	5.17	29.24	1.02	*t*(29) = −8.070, *p* < .001***	[−10.06, −5.99]
ACE/100	64.63	13.60	93.83	3.50	*t*(29) = −10.830, *p* < .001***	[−34.71, −23.69]
Semantic fluency	11.29	5.24	22.14	4.54	*t*(53) = −8.342, *p* < .001***	[−13.46, −8.24]
Phonemic fluency	6.75	3.25	14.17	4.16	*t*(53) = −7.523, *p* < .001***	[−9.40, −5.44]

GDS = Geriatric Depression Scale; MMSE = Mini Mental State Assessment; ACE = Addenbrooke’s Cognitive Examination; CI = confidence intervals. The MMSE is included in the ACE. Fluency scores are presented as the total number of items recalled. ***p < .001

### Materials

#### Neuropsychological assessment

Participants were assessed for global cognitive functioning with the Addenbrooke’s Cognitive Examination (ACE; Mathuranath et al., 2000), which includes the MMSE (Folstein et al., 1975). Higher scores on both measures indicate better cognitive ability. Participants were also assessed on phonemic fluency (letter *S*) and semantic fluency (animal category; Lezak et al., 2012) as part of the ACE. Presence of depressive symptoms were assessed with the Geriatric Depression Scale (GDS; Brink et al., 1982; Djernes et al., 2004), which is a self-report scale consisting of 15 items that can be answered with a yes or no in reference to how the respondent felt over the past week. Higher scores representing more depressive symptoms, with scores ≥6 being shown to be indicative of depression in a Danish validation study with frail elderly participants (Djernes et al., 2004).

#### The flashbulb memory questionnaire

The questions included in the questionnaire were based on earlier work (e.g., Brown & Kulik, 1977; Berntsen & Thomsen, 2005). A total of 18 events were included in the FBM questionnaire (see Table 2 and the Appendix). All events were covered by national media when they took place and were sufficiently surprising and consequential to be probable FBM events. The events were carefully selected in collaboration with two experts—a historian specialized in Danish 20th-century history and a social anthropologist and museum curator with extensive knowledge of Danish everyday life during the same historical period. For each decade, two events were included to increase the likelihood that participants were highly familiar with at least one of the two events. Moreover, this number of events was deemed adequate for generating a sufficient number of answers without fatiguing, especially the AD participants. The questionnaire was accommodated to each participant so that the events shown spanned from the first decade of their lives until the present (e.g., if a participant was born in 1939, the first events shown would be from the 1940s spanning to the 2010s). As such, a participant born in 1939 would be probed with a total of 16 events.

**Table 2:** List of events used to elicit flashbulb memories in chronological order

Decade	Nr.	Event
1930s	1.	Kanslergadeforliget, Denmark, January 30, 1933
	2.	Hindenburg disaster, USA, May 6, 1937
1940s	1.	The Danish occupation, Denmark, April 9, 1940
	2.	Denmark’s Liberation Day, Denmark, May 5, 1945
1950s	1.	The sinking of “Hans Hedtofts,” Denmark, January 30, 1959
	2.	The catastrophe on Haderslev Dam, Denmark, July 8, 1959
1960s	1.	Assassination of John F. Kennedy, USA, November 22, 1963
	2.	First human to step the moon (Neil Armstrong), USA, July 21, 1969
1970s	1.	Coronation of the Queen Margrethe II, Denmark, January 14, 1972
	2.	A referendum on joining the European Economic Community, Denmark, October 2, 1972
1980s	1.	Assassination of Oluf Palme, Sweden, February 28, 1986
	2.	Fall of the Berlin Wall, Germany, November 9, 1989
1990s	1.	Denmark wins gold at the European Football Championship, Sweden (Germany versus Denmark), June 17, 1992
	2.	Death of Diana, Princess of Wales, France, August 31, 1997
2000s	1.	September 11 Attacks, World Trade Center, USA, September 11, 2001
	2.	Indian Ocean earthquake and tsunami, Asia, December 26, 2004
2010s	1.	Krudttønden attack and the Great Synagogue Shooting, Denmark, February 14–15, 2015
	2.	Donald Trump is elected the president of USA, USA, November 8, 2016

The procedure adopted in the present study was an elaboration of Brown and Kulik’s (1977) original work (see also Berntsen & Thomsen, 2005). For each event probed, participants were presented with two to four photographs^1^ of the event on a 21.0 × 29.7-cm white card, while the interviewer named aloud the event. Participants were then asked to provide a memory that referred to the specific event following the instruction: “Do you recall where you were and what you were doing when you first learned that [the named event].” If participants answered yes, they were asked to describe in detail their personal memory of receiving the news. The participants, depending on their age and thus in which decade they were born, would be presented with a total of 14–18 events.

Given the study population, the questioning approach and timing had to be tailored to accommodate the needs and abilities of the participants. For that reason, the time frame was somewhat flexible in order to provide each participant with enough time to consider the request for a personal memory related to a particular event. However, if participants continued to provide only historical information about the named event or diverged from the topic, the interviewer would probe for a personal memory of receiving the news of the event. If it emerged that the participant was unable to recall a personal memory or did not contribute new information, the interviewer was instructed to gently stop the participant and proceed to the next event on the questionnaire. This was done to avoid exhausting participants.

The presentation of the events was counterbalanced so that half of the participants started with events from the 2010s going backwards to the decade the participant was born, whereas the other half were presented with the events in reverse order. If not automatically provided by the participants, the interviewer probed for additional details, such as how they first learned about the event, and what they were doing immediately before the event (see Table 3).

**Table 3:** Additional questions

1	When did you first hear about the event?
2	Where were you when you learned about the event?
3	Where the other people present, when you learned about the event?
4	What were you doing when you learned about the event?
5	What were you doing immediately before you learned about the event?
6	Do you remember any other special details from when you learned about the event?

When participants indicated the presence of a FBM, whey were furthermore asked to rate the degree to which they felt as though they were reexperiencing the event on a 5-point Likert scale (1 = *not at all* to 5 = *a high degree*). Answers generated in response to the FBM questionnaire were audio recorded to allow for subsequent transcription and coding of events.

### Procedure and scoring

All participants were tested individually in their own home by trained psychological staff. After obtaining informed consent, participants were assessed on the neuropsychological and clinical measures (MMSE, ACE, and GDS). Then the Galton–Crovitz word-cuing task was administered,^2^ followed by the FBM questionnaire. For most participants, all tasks were completed in one session; however, in a few instances, the experimenter administered the FBM task to the AD participants in a second session, due to participant exhaustion.

Participants’ verbatim responses to the FBM questionnaire were scored for presence of FBMs by two independent coders. The coders assessed (a) whether the participants themselves indicated that they had a FBM (*yes* or *no*; i.e., confirmed that they could remember *where they were and what they were doing* when receiving the news of the event) and (b) whether their description of the event included concrete information to substantiate the “where” and “what” (*yes* or *no*). In some instances, responses were scored as a FBM based on the details provided in the memory description, rather than on participants explicitly indicating that they had a FBM of the event. The two coders scored 20% of the transcripts independently. Their agreement rates were 88% for presence of FBM and 87% for presence of concrete information. Differences were resolved through discussion. One coder scored the remaining transcripts.

In order to examine the content of the reported memories, all event descriptions were also assessed for information on the following categories: (1) *time* (when the participant heard the news such as time of day, month), (2) *place* (where the participant was located when hearing the news), (3) *Informant (source)* (from who or what the participant heard the news), (4) *ongoing activity* (what the participant was doing when receiving the news), (5) *activity before* (the activity that the participant was engaged in immediately before the ongoing activity at the time of the news), (6) *own affect* (own emotional or physical reactions to hearing the news), (7) *others present* (other persons present when one received the news), (8) *affect in others* (how others felt or reacted when hearing the news), (9) *aftermath* (the immediate aftermath following the news of the event, including any consequences for the person), and (10) *other details* (any other distinctive details from the event). Six of the categories (2, 3, 4, 6, 8 and 9) were based on Brown and Kulik’s (1977) canonical categories found to be typical for FBMs. Four additional categories were added: *time*, *others present*, and *activity before*, which have previously been identified in the FBM literature (e.g., Berntsen & Thomsen, 2005; Kizilöz & Tekcan, 2013), while the category *other details* was included to allow for assessment of idiosyncratic details, such as descriptions of the weather, perceptual information, and thoughts. Each category was dichotomously scored as either present (scored as 1) or not present (scored as 0). The categories were summed for a total score ranging from 0 to 10 for each memory, reflecting the degree of detail and elaboration on each event. Interrater agreement based upon 20% of the transcripts ranged between 80–95% for information category, and the reliability coefficient was 0.92 for the composite score, as estimated by the intraclass correlation coefficient using a two-way random effects model (Shrout & Fleiss, 1979).

### Data analysis

Statistical analyses were performed using IBM SPSS statistics (Version 28) for Windows. Group differences were tested using Welch’s *t* test to account for unequal variance (Delacre et al., 2017), rounding the degrees of freedom to the nearest whole number. Pearson’s chi-squared tests were used to examine frequency patterns of memories across the AD and HC groups. Spearman’s correlations were conducted to explore associations between performance measures. We report Cohen’s *d* effect sizes to indicate the relative strength of significant group differences.

## Results

We first report results from the neuropsychological assessment. We next examine group differences regarding the frequency of FBMs and their phenomenological qualities. We then present findings on FBMs for the two groups as a function of age at the time of the event.

### Neuropsychological measures

As shown in Table 1, the AD group scored significantly lower on measures of cognitive functioning (MMSE and ACE) relative to the HC group. The AD participants were also impaired on executive functioning, as demonstrated by the phonemic and semantic fluency scores. There was also a trend towards a higher level of depressive symptoms in the AD group. However, the mean score of the AD participants (*M* = 2.42, *SD* = 2.34) was below the cutoff of the Geriatric Depression Scale (≥6).

### Frequency of flashbulb memories in response to probed events

Memory frequency for the circumstances around receiving the news of the probed events was assessed between groups. However, as the number of probed events differed across participants depending on their age (i.e., how many decades they had lived through), we calculated the percentage of events involving FBMs out of the total number of probed events for each participant.

As expected, the HC group reported recalling the circumstances around receiving the news for a significantly higher percentage of events (36.40%) relative to the AD group (16.11%), *t*(51) = −4.57, *p* < .001, *d* = −1.20. For percentage of FBMs that included concrete information on “where” and “what” out of the total number of probed events, a similar pattern emerged, with the HC’s providing concrete details on where they were and what they were doing when they heard the news for 36.40% of the events, relative to 16.71% of the events in the AD group, *t*(51) = −4.45, *p* < .001, *d* = −1.17. For the AD group, the slightly higher percentage of FBMs in the latter condition, reflected that 69 FBMs were identified from participants themselves indicating that they had a FBM and 73 FBMs were identified based on assessing whether descriptions contained concrete information about “where” and “what.” Only in one instance, were an AD participant not able to provide information about “where” and “what” after reporting having a FBM for the event. The clear correspondence between when participants themselves indicated that they had a FBM and their description containing concrete information to substantiate the “where” and “what” observed for both groups (98.6% and 100% in the AD and HC group, respectively), suggest that AD participants retained the ability to determine if they had a FBM for the probed events.

Table 4 shows the raw frequencies and percentages of participants reporting FBMs for the probed events. Although the percentage of participants reporting FBMs were lower in the AD group for most events, chi-squared tests revealed significant group differences in memory frequency only for five of the 18 events included in the FBM questionnaire.

**Table 4:** Number and percentage of participants reporting that they remembered when they first heard about the news

Event	Alzheimer		Control		
	*N*_FBM_/*N*_Probed_	%	*N*_FBM_/*N*_Probed_	%	χ^2^(1)
Kanslergadeforliget 1933	0/3	0.0	0/4	0.0	–
Hindenburg disaster 1937	0/7	0.0	0/4	0.0	–
The Danish occupation 1940	8/16	50.0	8/10	80.0	2.34
Denmark’s Liberation Day 1945	11/21	52.4	12/20	60.0	0.24
The sinking of Hans Hedtoft 1959	5/28	17.9	5/29	17.2	0.00
Haderslev Dam catastrophe 1959	1/28	3.6	3/29	10.3	1.00
Assassination of JFK 1963	7/28	25.0	21/29	72.4	12.81***
Moon landing 1969	4/28	14.3	13/29	44.8	6.35*
Coronation of Margrethe II 1972	1/28	3.6	6/29	20.7	3.88*
EEC referenda 1972	1/28	3.6	5/29	17.2	2.83
Assassination of Oluf Palme 1986	4/28	14.8	4/29	13.8	0.01
Fall of the Berlin Wall 1989	4/28	14.3	5/29	17.2	0.09
European Championship 1992	6/28	21.4	19/29	65.5	11.25***
Death of Princess Diana 1997	3/28	10.7	7/29	24.1	1.77
September 11 attacks 2001	10/28	35.7	24/29	82.8	13.10***
Tsunami in the Indian Ocean 2004	0/28	0.0	12/29	41.4	14.68***
Krudttønden attack 2015	3/28	10.7	9/29	31.0	3.54
Donald Trump elected president 2016	1/28	3.6	8/29	27.6	6.18*

Number of participants reporting FBMs for each individual event. Percentages represent the percentage of participants probed with the event in question who reported an FBM in response to the event probe. *p > .05, **p < .01, ***p < .001

The formation of FBMs is known to depend on the age of the participant at the time of the event. Previous studies have shown that the ability to form FBMs increases as a function of age up to the age of 8 years at the time of the event (Berntsen & Rubin, 2006; Winograd & Killinger, 1983), consistent with the literature on childhood amnesia. We therefore examined whether the frequency of FBMs increased as a function of age at the time of events up to age 8. Correlational analyses using Spearman’s rho revealed a positive correlation between having a memory and age at event, *r*(27) = .41, *p* = .034 in the HC group. This finding is consistent with an age-related increase in the ability to form FBMs during early childhood. In contrast, for the AD group, no significant correlation was found, *r*(28) = .10, *p* = .600.

Among HCs who were 8 years or older at the time of the Danish occupation (*n* = 4) and Denmark’s Liberation Day (*n* = 9), all reported a FBM for these two events, in agreement with previous work (Berntsen & Rubin, 2006; Berntsen & Thomsen, 2005). For the AD participants, who were a least 8 years old at the time of the events, 83.3% had a FBM of the Danish occupation (out of *n* = 6 participants) and 61.5% of Denmark’s Liberation (out of *n* = 13 participants).

### Group differences in category sum score

To examine whether memory of the reception context was preserved in AD, we compared the mean category sum score, reflecting the degree of detail and elaboration, across groups (including only self-reported FBMs). A significant difference was observed between the HCs (*M* = 5.73, *SD* = 1.24) and the AD participants (*M* = 4.52, *SD* = 1.38), *t*(48) = −3.35, *p* = .002, *d* = −.93. Hence, even for reported FBMs, AD participants’ performance was impaired compared with that of the HCs.

### Recall of information categories for flashbulb memories

To examine whether AD participants and HCs differed in the specific details recalled, we compared the mean percentages of each information category for remembered events across the two groups (see Table 5). Regarding Brown and Kulik’s (1977) original canonical categories, no significant differences were detected for *place, source*, *ongoing activity*, *own affect*, *affect in others*, or *aftermath*, suggesting that although AD participants’ memories were less detailed overall, the core features of their FBMs remained relatively preserved. For the four other categories, AD participants produced fewer events that included information about the *time* and *activity before* hearing about the event, while no significant differences were seen for *others present* or *other details* (see Table 5).

**Table 5:** Mean percentage of reported flashbulb memories that included details on each category of information

	Alzheimer		Control			
	*M*	*SD*	*M*	*SD*	*t*(*df*)	CI
**Brown and Kulik’s canonical categories**						
Place	75.27	37.04	90.80	15.70	*t*(32) = −1.95, *p* = .061	[−31.79, 0.74]
Informant (source)	81.33	34.80	95.92	9.35	*t*(27) = −2.03, *p* = .052	[−29.32, 0.14]
Ongoing activity	51.33	39.31	65.57	28.53	*t*(43) = −1.49, *p* = .143	[−33.46, 4.99]
Own affect	68.40	34.29	80.90	19.35	*t*(37) = −1.61, *p* = .116	[−28.25, 3.25]
Affect in others	34.93	35.38	46.63	35.57	*t*(50) = −1.20, *p* = .236	[−31.30, 7.90]
Aftermath	11.33	25.33	19.95	17.24	*t*(42) = −1.43, *p* = .160	[−20.79, 3.54]
**Other categories**						
Time	23.87	31.94	41.36	27.38	*t*(48) = −2.13, *p* = .039	[−34.03, −0.96]
Activity before	5.00	13.23	17.33	21.52	*t*(46) = −2.54, *p* = .015	[−22.10, −2.56]
Others present	66.87	36.94	77.57	25.98	*t*(43) = −1.21, *p* = .234	[−28.60, 7.19]
Other details	32.73	33.51	37.85	26.56	*t*(46) = −0.61, *p* = .544	[−21.97, 11.74]

Welch *t* test was used for the analysis

### Ratings of reexperience

No significant difference was seen for mean ratings of degree of reexperience of the remembered events for AD participants (*M* = 3.45, *SD* = 1.36) and HCs (*M* = 3.89, *SD* = 0.82), *t*(23) = −1.23, *p* = .231, *d* = −.43.

To examine whether degree of memory detail would result in higher reexperience, bivariate correlations were carried out in each group (under the assumption that these measures can be treated as independent observations). Spearman’s rho correlations revealed significant correlations between degree of detail (category sum score) and self-rated reexperience for both the AD participants, *r*(42) = .31, *p* =.046, and the HCs, *r*(148) = .54, *p* < .001.

### Characteristics of FBMs for high-profile events

Some events were much more likely to be associated with FBMs than were others, with the five most frequently remembered events being the same across the two groups (see Table 4). These were the *Danish occupation*, *Denmark’s Liberation Day*, the *Assassination of JFK*, *Winning the European Championship*, and the *September 11 attacks*, showing that some public events were more likely to create FBMs.

We compared the phenomenological qualities of FBM for these high-profile events across the two groups. Here, significant differences in the category sum score were found for the Danish occupation, Denmark’s Liberation Day, and the September 11 attacks with AD participants providing fewer details. However, for the two other events, no significant differences were detected, and no significant differences were found for degree of reexperience between the two groups for any of the high-profile events (see Table 6). This suggests that deficits in the phenomenological aspects of FBMs varied depending on the concrete event being examined.

**Table 6:** Descriptive Statistics and t-test results for the category sum score and reexperience for the FBM questionnaire

	Alzheimer			Control			*t*(*df*)	95% CI
	*n*	*M*	*SD*	*n*	*M*	*SD*		
**Category sum score**								
Danish occupation	8	5.25	1.04	8	6.63	0.92	*t*(14) = −2.81, *p* = .014	[−2.42, −0.33]
Liberation day	11	5.27	1.74	12	8.08	1.16	*t*(17) = −4.52, *p* < .001	[−4.12, −1.50]
Assassination of JFK	7	5.00	1.83	21	6.00	2.12	*t*(12) = −1.20, *p* = .252	[−2.81, 0.81]
European Championship	6	5.33	1.86	20	5.63	2.48	*t*(11) = −0.31, *p* = .759	[−2.38, 1.79]
September 11 attacks	10	5.00	2.36	24	6.88	2.55	*t*(18) = −2.13* p* = .049	[−3.74, −0.01]
**Reexperience**								
Danish occupation	4	4.00	2.00	8	4.25	1.49	*t*(5) = −0.22, *p* = .834	[−3.20, 2.70]
Liberation day	6	3.67	1.51	12	4.42	0.79	*t*(6) = −1.14, *p* = .294	[−2.33, 0.83]
Assassination of JFK	6	4.17	1.60	21	3.95	1.28	*t*(7) = 0.30,* p* = .772	[−1.47, 1.90]
European Championship	5	3.20	1.64	17	3.76	1.20	*t*(5) = −0.71, *p* = .505	[−2.56, 1.43]
September 11 attacks	6	4.50	0.84	22	4.36	0.79	*t*(8) = 0.36,* p* = .730	[−0.75, 1.02]

Welch *t* test was used for the analysis

### Effects of lifetime period on flashbulb memories

**Fig. 2:**
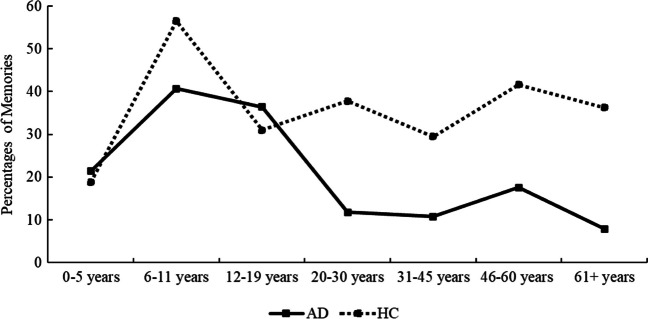
Percentage of flashbulb memories across the five life-time periods for the AD and HC group

To examine the temporal distribution of FBMs across the life span, all events were pooled across participants in each group. The AD group provided 69 memories out of the 438 that were possible if all AD participants had reported a memory in response to every event probe, whereas the HCs provided 161 memories out of 444 possible reports. Figure 2 shows the frequencies across seven lifetime periods: 0–5 years of age, 6–11 years of age, 12–19 years of age, 20–30 years of age, 31–45 years of age, 46–60 years of age, and 61 years of age and up, which are roughly consistent with the time bins used in prior work examining the temporal distribution of autobiographical memories (e.g., Barnabe et al., 2012; Kirk & Berntsen, 2018).

As illustrated by Fig. 2, FBMs were clearly not evenly distributed across the seven lifetime periods in the AD group, χ^2^(6) = 31.52, *p* < .001, with participants showing a dominance of FBMs from age 6 to 19 followed by a steep drop. In contrast, no significant effect of life period on recall of FBMs was observed for HCs, χ^2^(6) = 10.23, *p* = .115, although the distribution did indicate a peak for the 6-11 years of age time bin (see Fig. 2).

There were no significant group differences in the frequency of reported FBMs from 0 to 19 years of age, indicating that FBMs from these periods were relatively preserved in AD (*p*s > .266). In contrast, significant group differences were found for the four most recent time bins, with the AD group performing significantly worse relative to HCs (*p*s < .01; see Table 7).

**Table 7:** Percentage of reported FBMs across the seven lifetime periods on the FBM questionnaire

	Alzheimer		Control		
	*n*	*%*	*n*	*%*	χ^2^(1)
**Frequency**					
0–5 years	3	21.43	3	18.75	0.03
6–11 years	11	40.74	13	56.52	1.24
12–19 years	12	36.36	13	30.95	0.24
20–30 years	9	11.84	31	37.80	14.06***
31–45 years	9	10.84	22	28.21	7.80**
46–60 years	16	17.58	42	41.58	13.08***
61+ years	9	7.89	37	36.27	25.87***

**Fig. 3:**
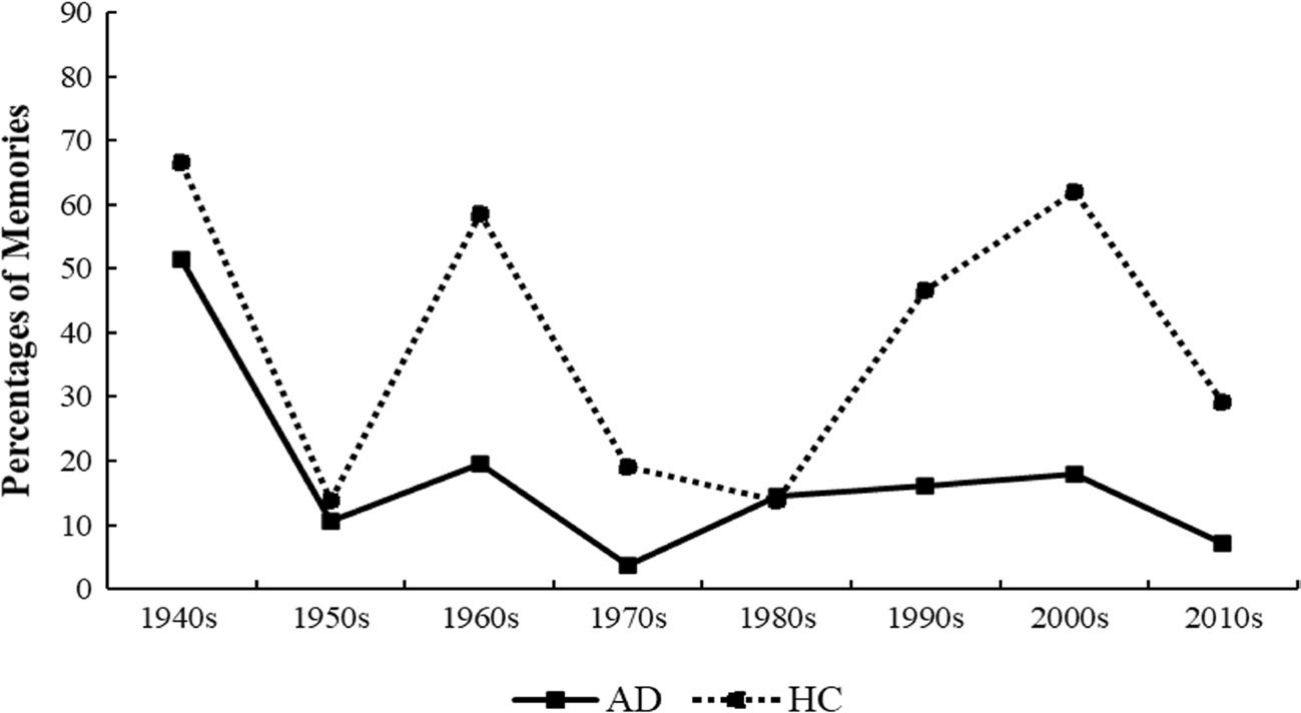
Distribution (in percentages) of flashbulb memories across decades. *Note.* The peak in the 1940s reflects the two WWII events (the Danish occupation in 1940 and Liberation Day in 1945); the peak in 1960s reflects the assassination of JFK in 1963; and the peak across the 1990s and 2000s reflects Denmark Winning the European Championship in 1992 and the terrorist attacks of 11 September 2001

However, as certain events were more likely than others to generate FBMs (i.e., high-profile events), we also plotted the FBMs chronologically by the decade of their occurrence. As illustrated in Fig. 3, the distribution was characterized by three spikes in FBMs: the 1940s corresponding to both of the WWII events (the Danish occupation in 1940 and Liberation Day in 1945), which were highly distinctive for both groups, in line with these events being particular accessible; the 1960s corresponding to the assassination of JFK; and the 1990s and 2000s corresponding to Denmark Winning the European Football Championship and the September 11 terrorist attacks, which were more pronounced in the HC group. This indicates that retrieval was, at least in part, driven by the memorability of the specific public events, rather than only by participants age at the time of the event.

## Discussion

The aim of the present study was to examine memory for the reception of important public events across the life span in individuals with AD, compared with healthy older adult. The results of our study demonstrated that FBM was impaired in AD, with this group reporting significantly fewer FBMs and providing less contextual detail overall compared with HCs. However, similarities were also observed across the two groups. First, AD participants and HCs showed a similar pattern of performance, the most frequently remembered events being the same across groups. Second, despite AD participants reporting fewer details overall, no significant group differences were found for most content categories, suggesting that people with AD do retain some memory of the reception context for important public events. Importantly, our results showed that FBM performance in AD varied depending on the specific lifetime period and specific event being assessed.

### Frequency of flashbulb memories

In line with previous work concerning autobiographical memory for emotional events (Philippi et al., 2015), AD participants demonstrated a quantitative deficit, recalling fewer FBMs overall. However, this effect varied depending on the specific events and lifetime period being probed, with no differences being observed for FBMs dated to the period between 0 and 19 years of age (see Fig. 2). A similar pattern, with no deficits in memory frequency for the early years (0–15 years of age), was also reported in a study on life story memories in AD (Rasmussen & Berntsen, 2023), and accords with general observations that AD involves a relative sparing of memories for the remote past (e.g., Barnabe et al., 2012; Graham & Hodges, 1997; Greene et al., 1995; Kirk & Berntsen, 2018). Our study expands these findings on retrograde amnesia in AD by showing that the relative sparing of early memories also applies to FBMs.

AD participants showed a predominance of FBMs from the 6–19-year period followed by a steep drop. This pattern has some overlap with the temporal distribution observed in previous studies (e.g., Berntsen et al., 2022; Fromholt & Larsen, 1991; Rasmussen & Berntsen, 2023) and may be indicative of an early reminiscence bump (Rubin et al., 1998; Rubin & Schulkind, 1997). It is also consistent with findings from the collective memory literature showing some evidence that memory for important public events is most pronounced for events occurring during ones youth (Schuman & Scott, 1989; but see Koppel, 2013; Koppel & Berntsen, 2016, for more mixed results). No effect of lifetime period was found on FBM frequency in the HCs, except for an increase in FBMs up to the age of 8, consistent with a period of childhood amnesia. However, HCs did show a trend towards a bump for the 6 to 11 years of age time bin (see Fig. 2), suggesting that events from this period were more frequently recalled, in line with prior findings of a reminiscence bump in FBMs in healthy aging (e.g., Denver et al., 2010). Interestingly, a similar pattern, characterized by a bump in the AD group and the absence of such a bump in HCs, was also reported in a recent study that explored autobiographical recall across seven lifetime periods (Berntsen et al., 2022). This suggests that the cueing technique of probing events from specific lifetime periods, as opposed to freely across the life span, may partially account for our findings. Furthermore, factors related to collective remembering, such as perceived historical relevance, are likely to have also formed memories at the individual level.

Overall, these findings do not support a flat gradient, as predicted by the multiple trace theory, or a clear temporal gradient with a monotonic decrease, consistent with consolidation theory. The sparring of FBMs from childhood and adolescence followed by a marked decline in frequency may be accounted for by a reminiscence bump in AD; however, it may also be that event specific characteristics made FBMs from this period more accessible. Specifically, the World War II events from the 1940s were particularly accessible to both groups. These events serve an important role for Danish national identity and have been much commemorated and publicly rehearsed over the years, which may have influenced memory consolidation and maintenance processes (Berntsen, 2009).

### Phenomenological qualities of flashbulb memories

The results concerning memory for contextual details were less clear. Only 2 out of 10 categories revealed a statistically significant group difference (details on time and activity before) with no differences being found for any of Brown and Kulik’s (1977) canonical categories. That is, for the FBMs AD participants did produce, they were able to provide information about the content (e.g., where they were, how they first became aware of the event, what they were doing, how they felt or reacted, how others reacted and the immediate aftermath following the event), at a level comparable with HCs. These findings suggest that while their FBMs were less detailed overall (based on the category sum score), the core characteristics of FBMs were retained in AD. This contrasts with the compromised retrograde memory for contextual details normally reported in individuals with AD (e.g., Greene et al., 1995; Ivanoiu et al., 2006; Leyhe et al., 2009) and suggests that FBMs have a positive effect on retrieval in the case of AD, in line with findings from studies focusing on anterograde memory in AD (e.g., Çebi et al., 2020; El Haj et al., 2016; Ikeda et al., 1998). These results may reflect some residual emotional enhancement effect in AD (e.g., Philippi et al., 2015). However, other processes such as enhanced rehearsal likely also contribute (e.g., Müller et al., 2016). This is consistent with evidence that frequently rehearsed memories for public events are better preserved in AD, regardless of the remoteness of these events (Langlois et al., 2016).

Interestingly, AD participants’ ability to report FBMs varied depending on the specific event being probed, with no significant group differences being detected on level of detail (the category sum score) for two of the five high-profile events (i.e., the assassination of JFK and Denmark winning the European Championship). This is important because most studies have assessed FBM in AD, focusing on a single event taking place after dementia onset. The present finding suggests that differences in memorability of the public events may help account for some of the divergent results regarding FBMs previously reported in AD.

Also, in contrast to general findings of compromised episodic reliving in AD (see El Haj et al., 2015, for a review), no group differences were found for degree of reexperiencing the retrieved FBMs. This finding fits with the results of a case study on anterograde FBM in an individual with mild AD, who reported a high degree of mental time travel and mental imagery for the Paris attacks in 2015 (El Haj et al., 2016). Here, we showed that this finding extends to retrograde retrieval of FBMs, and that this sustained subjective reliving is consistent across time and events. The validity of these findings was further supported by degree of reliving being positively correlated with number of contextual details both in HCs and AD participants.

### Limitations

The present study has limitations. First, because this study examined memories related to real-life public events that happened at specific times in history and necessarily varied regarding perceived importance both nationally and internationally, it was not possible to fully match events on characteristics such as consequentiality, nationality, salience, or emotional valence. Still, all included events received extensive media coverage after they took place and are the kind of consequential and surprising events that FBMs typically are made of, according to independent historical assessments. Second, a unique strength of the present study was the systematic examination of FBMs across the life span of the participants, and the inclusion of both older adults with AD and older healthy adults, none of which has been done before. Yet, because the participants did not have the same exact age at the time of testing, and because of the memory deficits in the AD group, the sample of events that each participant could meaningfully respond to necessarily varied between participants. Third, the design of the study did not include a comparison or control memory, such as a non-FBM from each decade, because it is unclear exactly what such condition should be and because such inclusion would have rendered the study considerably more time consuming and involved a real risk of exhausting the AD participants. However, the fact that the temporal distribution of the FBMs mimics that of earlier work examining important personal events (e.g., Berntsen et al., 2022; Fromholt & Larsen, 1991; Rasmussen & Berntsen, 2023) supports the validity of a reminiscence bump in AD. Moreover, prior studies examining important personal events have also reported that declining memory frequency was not accompanied by less detailed or specific events in AD (e.g., Fromholt & Larsen, 1991; Rasmussen & Berntsen, 2023), suggesting that perceived importance or emotionality is associated with enhanced memory in AD. Fourth, given the central role of rehearsal in FBM formation and maintenance, further studies could consider to include measures assessing rehearsal, including examining the impact of commemorations on FBM retrieval in AD, although it may be difficult to meaningfully obtain such retrospective assessments given the memory deficits in the population. Fifth, the small number of FBMs collected from some time periods means that any null findings regarding lifetime periods or events should be interpreted with caution. Finally, we do not know when AD participants’ memory impairments began, which means that FBMs related to some of the later public events may have been influenced by anterograde amnesia rather than only reflecting deficits in retrograde recall.

## Conclusion

Overall, AD participants were able to report FBMs, but the frequency of reported FBMs was lower than for HCs. Importantly, despite such quantitative differences, our findings suggest that qualitative aspects of AD participants’ FBMs were quite similar to those of healthy older adults, as no significant group differences were obtained with respect to the canonical categories or the subjective degree of reexperience for memories reported. This suggests that people with AD can retain quite detailed episodic memories over long temporal delays, when cued by requests for FBMs. AD participants’ FBMs clustered during the early years of their life, whereas HCs also reported FBMs dated to later lifetime periods. The findings suggest that probing for personal memories of important public events may serve as a powerful cue for detailed episodic memories in AD, which may help maintain a sense of personal and social identity.

## Appendix. Description of the events probed in the FBM questionnaire

- **Kanslergadeforliget, Denmark, January 30, 1933:** An important political agreement in Denmark that shaped economic policies and laid the foundation for the Danish welfare state. It received notable media coverage at the time.
- **Hindenburg disaster, USA, May 6, 1937:** The catastrophic explosion of the German airship Hindenburg. It had substantial international impact, so Danish media also covered it extensively.
- **The Danish occupation, Denmark, April 9, 1940:** Denmark was occupied by Nazi Germany during World War II. This event received extensive media coverage as a pivotal moment in Danish history.
- **Denmark’s Liberation Day, Denmark, May 5, 1945:** Denmark’s liberation from German occupation in World War II. Received significant coverage as a pivotal moment in Danish history.
- **The sinking of “Hans Hedtofts,” Denmark, January 30, 1959:** The sinking of a Danish passenger ship in the North Atlantic, claiming 95 lives. Received extensive coverage in Danish media due to the national tragedy.
- **The catastrophe on Haderslev Dam, Denmark, July 8, 1959:** A tourist boat exploded and burned up, claiming 57 lives. A national tragedy which received notable national coverage.
- **Assassination of John F. Kennedy, USA, November 22, 1963:** The assassination of the U.S. President. Given its global impact, Danish media covered it extensively.
- **First human to step on the moon (Neil Armstrong), USA, July 21, 1969:** A historic milestone in human space exploration. Received widespread global coverage, including in Danish media.
- **Coronation of Queen Margrethe II, Denmark, January 14, 1972:** The coronation of Denmark’s Queen Margrethe II. Received substantial coverage due to its national significance.
- **Referendum on joining the European Economic Community, Denmark, October 2, 1972:** Denmark’s referendum on joining the EEC. Received significant attention due to its implications for Denmark’s future.
- **Assassination of Oluf Palme, Sweden, February 28, 1986:** The assassination of Sweden’s Prime Minister. Received notable coverage in neighboring Denmark due to the proximity and significance of the event.
- **Fall of the Berlin Wall, Germany, November 9, 1989:** The collapse of the Berlin Wall symbolizing the end of the Cold War. Received widespread global coverage, including in Danish media.
- **Denmark wins gold at the European Football Championship, Sweden (Germany versus Denmark), June 17, 1992:** A significant sporting achievement for Denmark. Received substantial coverage in Danish media.
- **Death of Diana, Princess of Wales, France, August 31, 1997:** The tragic death of Princess Diana. Received extensive global media coverage, including in Denmark.
- **September 11 Attacks, World Trade Center, USA, September 11, 2001:** Terrorist attacks on the Twin Towers. Received widespread global coverage, including Danish media.
- **Indian Ocean earthquake and tsunami, Asia, December 26, 2004:** Catastrophic natural disaster resulting in a devastating tsunami. Received significant international coverage, including in Danish media.
- **Krudttønden attack and the Great Synagogue Shooting, Denmark, February 14–15, 2015:** Terrorist attacks in Copenhagen. Received substantial coverage in Danish media.
- **Donald Trump is elected president of the USA, November 8, 2016:** The election of Donald Trump as U.S. president. Received substantial coverage in Danish media due to its global implications and interest in U.S. politics.

## Footnotes

1 Photographs were chosen from Google.

2 Data from the Galton-Crovitz word-cuing task will be reported elsewhere.

## Declarations

### Open practices statement

Given patients’ sensitive information, and General Data Protection Regulations (GDPR), the database and codes generated during the current study are not publicly archived. However, access to materials will be granted from the corresponding author upon request but may require the completion of a formal data sharing agreement, in compliance with GDPR and Aarhus University rules. The study was not preregistered.
